# END-OF-LIFE DOULAS: EXPLORATION OF VALUES, GOALS, AND MOTIVATIONS

**DOI:** 10.1093/geroni/igad104.3301

**Published:** 2023-12-21

**Authors:** Amber Thompson

**Affiliations:** University of Utah, Salt Lake City, Utah, United States

## Abstract

End-of-life doulas provide non-medical support to individuals and families during end-of-life. Current research focuses on identifying the doula role and services offered at end-of-life, primarily in relation to existing medical care. The findings highlight the ambiguity of the role and resulting confusion for healthcare providers, individuals, and families. Using data collected from on-line surveys, the aim of this study was to explore the values, goals, and motivation of those identifying as end-of-life doulas (n=232). In addition to questions about the role and services provided, 14 items assessed the doulas’ underlying values through goals and motivations. Questions addressed their motivation for becoming a doula and their goals for this role, such as: 1) promoting well-being during end-of-life 2) providing a less medicalized model, including advocating for death reform, and 3) wanting to professionalize and/or commodify the non-medical care they provide. Exploratory factor analysis supports two underlying factors describing the doulas’ goals and motivations including social values (i.e., perceived positive impact on society) and functional values (perceived problem solution) (Cronbach’s alpha .85 and .75 respectively). Content analysis of open-ended survey responses describing motivations for becoming an end-of-life doula strengthen this perspective and expand it to include emotional motivations (i.e., “called” to the work, or personal experiences with death). These unique findings demonstrate the need for more research documenting the underlying values organizing end-of-life doulas. The potential benefits of investigating values include progress towards clarification about the role of end-of-life doulas, and how they may contribute to providing person-and-family centered care during end-of-life.

